# Dynamic Ultrasound Assessment of Median Nerve Mobility Changes Following Corticosteroid Injection and Carpal Tunnel Release in Patients With Carpal Tunnel Syndrome

**DOI:** 10.3389/fneur.2021.710511

**Published:** 2021-08-27

**Authors:** I-Ning Lo, Po-Cheng Hsu, Yi-Chao Huang, Chih-Kuang Yeh, Yi-Chiang Yang, Jia-Chi Wang

**Affiliations:** ^1^Department of Orthopaedics and Traumatology, Taipei Veterans General Hospital, Taipei, Taiwan; ^2^Department of Orthopaedics, National Yang-Ming University, Taipei, Taiwan; ^3^Department of Physical Medicine and Rehabilitation, National Taiwan University Hospital, Bei-Hu Branch, Taipei, Taiwan; ^4^Institute of Clinical Medicine, National Yang-Ming University, Taipei, Taiwan; ^5^Department of Physical Medicine and Rehabilitation, School of Medicine, National Yang Ming Chiao Tung University, Taipei, Taiwan; ^6^Department of Biomedical Engineering and Environmental Sciences, National Tsing Hua University, Hsinchu, Taiwan; ^7^Department of Physical Medicine and Rehabilitation, Taipei Veterans General Hospital, Taipei, Taiwan

**Keywords:** carpal tunnel release, carpal tunnel syndrome, dynamic ultrasound, nerve mobility, ultrasound-guided injection

## Abstract

Decreased median nerve (MN) mobility was found in patients with carpal tunnel syndrome (CTS) and was inversely associated with symptom severity. It is unclear whether MN mobility can be restored with interventions. This study compared the changes in MN mobility and clinical outcomes after interventions. Forty-six patients with CTS received an injection (*n* = 23) or surgery (*n* = 23). Clinical outcomes [Visual Analogue Scale; Boston Carpal Tunnel Questionnaire (BCTQ), which includes the Symptom Severity Scale and Functional Status Scale; median nerve cross-sectional area; and dynamic ultrasound MN mobility parameters (amplitude, and R^2^ value and curvature of the fitted curves of MN transverse sliding)] were assessed at baseline and 12 weeks after the interventions. At baseline, the BCTQ-Functional Status Scale and median nerve cross-sectional area showed significant inter-treatment differences. At 12 weeks, both treatments had significant improvements in BCTQ-Symptom Severity Scale and Visual Analogue Scale scores and median nerve cross-sectional area, but with greater improvements in BCTQ-Functional Status Scale scores observed in those who received surgery than in those who received injections. MN mobility was insignificantly affected by both treatments. The additional application of dynamic ultrasound evaluation may help to discriminate the severity of CTS initially; however, its prognostic value to predict clinical outcomes after interventions in patients with CTS is limited.

## Introduction

Carpal tunnel syndrome (CTS), the compression neuropathy of the median nerve (MN) at the wrist, is the most prevalent type of peripheral entrapment, causing pain, numbness, and tingling of the hand followed by weakness and muscle atrophy. As a result, it is recognized as an important cause of work-related disability (1). Treatment of CTS varies, ranging from conservative treatments to surgery. Ultrasound-guided corticosteroid injection has been shown to be effective for symptom relief (2, 3). Carpal tunnel release (CTR) has been proven to be a useful treatment in patients with severe CTS, and the long-term outcomes are favorable (4).

The widely proposed mechanism for the development of CTS is compression by the flexor retinaculum, causing elevation of intracarpal pressure and progressive neural ischemia, inflammation, and non-inflammatory synovial fibrosis (5, 6). MN swelling and increased cross-sectional area (CSA) are well-studied phenomena that serve as diagnostic criteria (7, 8). Unlike with CSA, there has been a lack of consensus in its application in nerve mobility due to the complexity of dynamic analysis. Nerve mobility is an adaptive response to dispersed stress during motion (9), and previous studies found decreased MN mobility in patients with CTS using dynamic ultrasound analysis (10–13). The severity of CTS has been shown to correlate with MN mobility (11, 12, 14).

The parameters that could be used objectively for assessing post-intervention improvements require investigation. Decreased MN CSA was observed in both interventions (13, 15); however, it is unknown whether different treatment approaches have similar effects with respect to MN mobility. Previous studies have evaluated the MN mobility change after surgery and found a tendency of increased displacement and changes in movement patterns after CTR (10, 16, 17). Our preliminary study demonstrated that the application of an anti-adhesion gel had no impact on MN mobility after CTR regardless of the improvement in clinical outcomes (18). Moreover, Moon et al. found no significant change in MN mobility after steroid injection in the early stage of CTS (19). We hypothesized that MN mobility would improve after both injection and surgery, indicated by MN CSA. Hence, we conducted a study to evaluate MN mobility and clinical outcomes in patients with CTS after either intracarpal injection or surgical intervention.

## Materials and Methods

### Study Design

This was a prospective, non-blinded study conducted at a single Medical Center between May 2016 and March 2019. Patients with a confirmed diagnosis of CTS by electrodiagnosis who had decided to undergo ultrasound-guided corticosteroid injection or CTR were asked to enroll in this study. All participants provided informed consent before enrolling in the investigation. An investigator collected medical history, carried out a physical examination, conducted electrodiagnostic tests, and administered questionnaires at the time of recruitment. The study was approved by the local institutional review board.

### Inclusion and Exclusion Criteria

Inclusion criteria for the present study were as follows: (1) age over 20 years; (2) typical CTS symptoms, including numbness in the sensory distribution of the MN; nocturnal, postural, or motion-associated paraesthesia ± pain in the subject's hand that is relieved by a flicking movement of the hand; (3) a positive Phalen test and/or presence of the Tinel sign; (4) persistent symptoms for more than 3 months; and (5) electrophysiologically confirmed median neuropathy at the wrist, regardless of the degree of severity.

Exclusion criteria of the study were as follows: (1) conditions that mimic CTS, such as cervical radiculopathy, polyneuropathy, brachial plexopathy, and thoracic outlet syndrome; (2) underlying disease such as diabetes mellitus, thyroid disease, wrist osteoarthritis, chronic renal failure on hemodialysis, autoimmune diseases, or pregnancy; (3) recent corticosteroid injection into the carpal tunnel within six months or previous CTR; (4) allergy to corticosteroids or local anesthetics; and (5) impaired cognitive function.

### Ultrasound-Guided Injection

An experienced physician performed the injection on participants using a Siemens 7–14-MHz linear array probe and the ACUSON SC2000 system (Siemens Medical Solutions, Mountain View, CA, USA). The probe was placed at the level of the proximal carpal inlet (scaphoid-pisiform plane) to identify the MN. After sterile preparation and using the in-plane ulnar approach, a 2-ml perineural injection with 1 ml of 2% lidocaine hydrochloride (xylocaine) and 1 ml of 40 mg triamcinolone acetonide was administered to the MN.

### Endoscopic Surgery

An orthopedic surgeon performed two-portal endoscopic CTR surgery under local anesthesia (20). One portal was in the mid-palm with direction to the ring finger, and the other was on the ulnar side of the palmaris longus tendon at the level proximal to the palmar crease. The transverse carpal ligament was completely released.

Patients who underwent CTR were followed up 1 week after surgery to remove stitches as well as to ensure integrity and healing of the surgical wound. Further follow-up of these patients was performed to evaluate surgical complications and improvement of symptoms. Dynamic ultrasound parameters and clinical outcomes were assessed 12 weeks after surgery.

### Primary Outcomes

Changes in the morphology and mobility of the MN were the primary outcomes of the study. The changes were assessed using a linear probe (5–14 MHz), the same ultrasound equipment used for the ultrasound-guided injection, which was placed transversely over the inlet of the carpal tunnel.

#### Median Nerve Cross-Sectional Area

The CSA was measured statically at the level of the carpal tunnel inlet. The patients were asked to position the forearm supinely and the wrist in the neutral position. The probe was placed without applying additional pressure to avoid MN deformity. The angle of incidence was adjusted by tilting or rotating the probe, to make the ultrasound beam perpendicular to the MN to achieve the highest resolution. The CSA of the MN was measured by continuous boundary tracing within the hyperechoic rim and calculated using the built-in software of the ultrasonography device. The measurements were repeated three times, and the mean value was used for analysis.

#### Median Nerve Mobility

MN mobility was measured using the method described by Kuo et al. (12). By using the ACUSON SC2000 system (Siemens Medical Solutions, Mountain View, CA, USA), the system settings including focus depth and time gain compensation were the same for all the participants.

**Figure 1:**
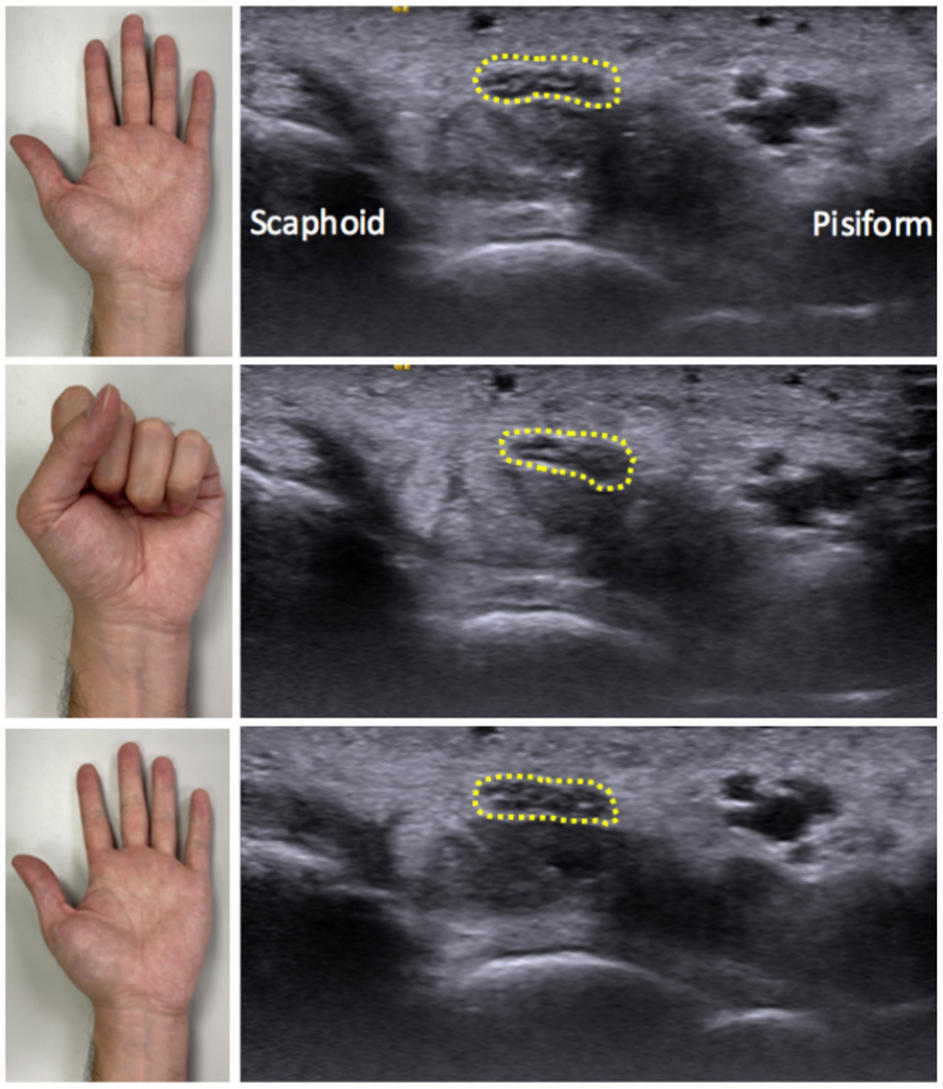
Illustration of wrist positioning during dynamic ultrasound scanning and the correspondingly ultrasound images. The participants were instructed to fully flex their hand from the initial neutral extension and then return to the beginning posture. The ultrasound probe was placed at the level of the carpal tunnel inlet, allowing the median nerve (dotted round) to be visualized.

All participants were asked to place their hand and forearm neutrally on the examination plate. The probe was gently placed at the inlet of the carpal tunnel without additional pressure, and the angle of incidence was adjusted perpendicular to the MN. After appropriate positioning, the participants were instructed to fully flex (clenched-fist posture) their fingers from the initial neutral extension and return to the initial posture (Figure 1). The flexion–extension cycle was executed within 3 s, and a dynamic video that clearly recorded the MN displacement was obtained for further analysis. The measurements were repeated three times and the mean value of the variables was recorded.

The speckle-tracking algorithm was used to analyze the MN displacement in the dynamic video during the flexion–extension cycle. A total of 36–40 frames of image were retrieved through the videos. As per the method proposed by Kuo et al. (12), the MN serves as the target region of interest, while the multilevel block-sum pyramid (BSP) algorithm (referred to as multilevel block-matching and BSP integrated algorithm) was applied for speckle tracking. First, the region of interest was determined by outlying the outermost hyperechoic rim of the MN manually in each frame.

**Figure 2:**
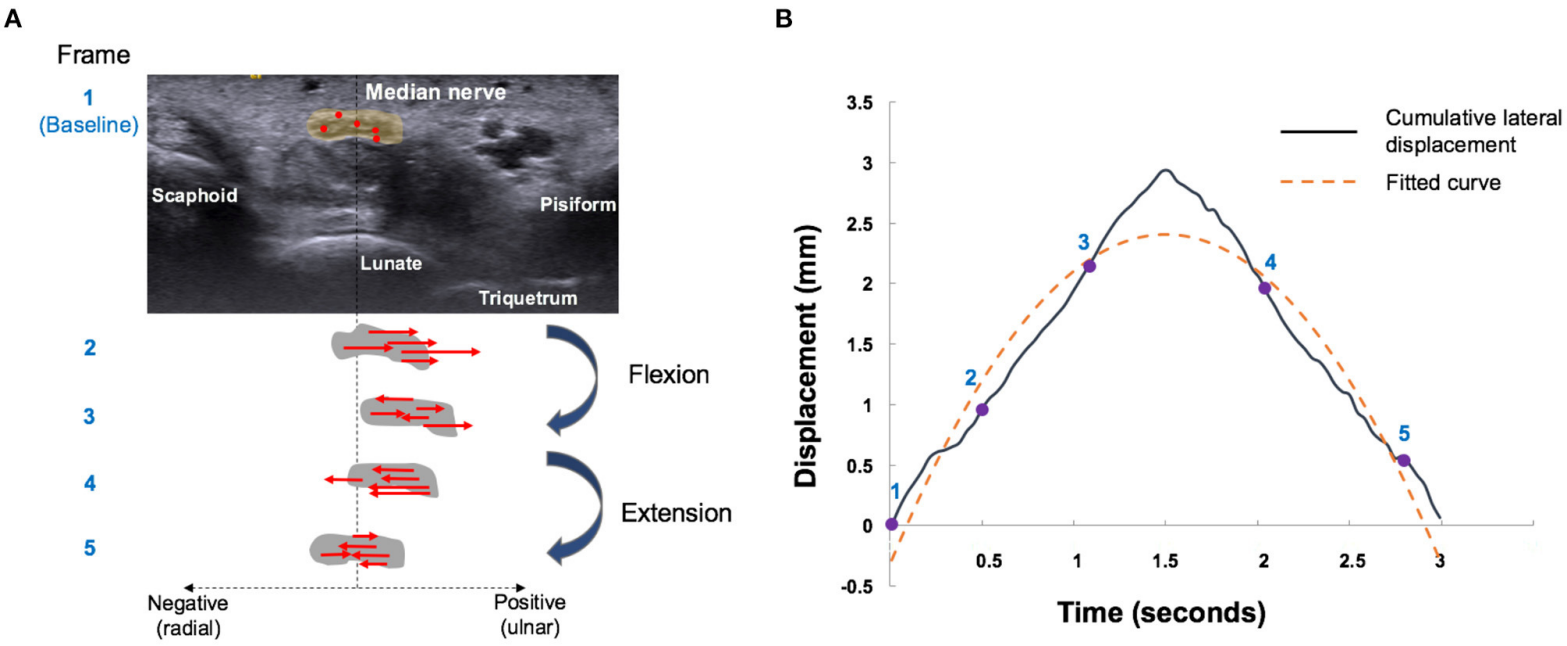
Median nerve motion pattern during finger flexion and extension movements. **(A)** Schematic five-frame drawing of speckle tracking algorithm of the median nerve (gray circle). Red dots indicate the representative pixels and red arrows indicate the direction of radial (negative) or ulnar (positive) displacement of each pixel by automatically matching between temporally close frames. Sum displacement of all the pixels in the median nerve boundary was calculated as the lateral displacement. The median nerve movements were tracked by repeating the aforementioned operation. **(B)** Radial-ulnar displacements (black curves) of the median nerve versus time were obtained by cumulating the lateral displacement during a single flexion–extension cycle. The number labels on the curve represent the corresponding points on the graph. The dotted yellow curve represents the fitted curve obtained by the second-order polynomial function [f(*x*) = a*x*^2^ + b*x* + c]. The curvature of the fitted curve and R^2^ were further calculated.

The multilevel BSP process consists of a repeated matching and searching process. The matching block in the reference image was set by a region of 32 × 32 pixels (26.9 pixels/mm), to compare with the test block of the comparison image. A 21 × 21 pixel block, which contained at least 10 speckles, was used as a search block, to define the best-matched pixel between two images. Displacement of each pixel was estimated by the calculating the distance between the matched pixel in the comparison image and the matching image. Lateral displacement was calculated as the sum displacement of all the pixels in the median nerve boundary between sequential images (Figure 2A). Ulnar and radial directions of the MN during the flexion–extension cycle were defined as positive and negative displacements, respectively (10, 12).

Cumulative lateral displacements versus acquisition time during the flexion–extension cycle can be curve-fitted by a second-order polynomial function [f(*x*) = a*x*^2^ + b*x* + c], which could be regarded as the sliding pattern of the MN (Figure 2B). Three parameters were further analyzed to describe the transverse sliding pattern of the MN: amplitude, curvature, and R^2^. Amplitude estimates represent the maximal transverse displacement of the MN. Curvature and R^2^ were derived from the fitting curve of the cumulated lateral displacement. Curvature estimates can be regarded as the functional compliance or elasticity of the MN in response to the flexion–extension cycle. R^2^ estimates represent the goodness of fit and were used to describe the transverse sliding function or smoothness of the MN in the flexion–extension cycle. These parameters were developed and used to differentiate CTS severity (12), while CTS severity was negatively correlated with amplitude, curvature, and R^2^.

### Secondary Outcomes

#### Visual Analogue Scale

This 11-point scale, which ranged from 0 (no pain) to 10 (worst pain imaginable), was used to assess the average level of pain, numbness, and paraesthesia over the wrist-hand region in the week prior to the assessment.

#### Boston Carpal Tunnel Syndrome Questionnaire

The Boston Carpal Tunnel Syndrome Questionnaire (BCTQ), which comprises two subscales [11 questions on Symptom Severity Scale (SSS) and eight questions on the Functional Status Scale (FSS)], was used to evaluate the symptom severity and functional status of the enrolled participants. Each question contained a five-point score; and the SSS and FSS scores were calculated as the means of the point retrieved on an individual scale. The BCTQ has shown good reliability and validity when applied in patients with CTS (21).

### Sample Size

No previous studies have evaluated MN mobility changes after intervention using the present quantitative method. Based on our preliminary unpublished data, a difference of 0.8 mm in amplitude with a standard deviation of 0.95 was observed at the 3-month follow-up after injection. The alpha level (α) was set to 0.05, and a power (β) of 80%; therefore, the estimated sample size was at least 19 participants in each study arm. The decision was to use 23 patients for each treatment, assuming that there would be a 20% dropout rate.

### Statistical Analysis

Baseline characteristics were analyzed by descriptive statistics, and continuous variables were expressed as mean ± standard deviation (SD). The chi-square test was used to compare categorical variables. The Shapiro–Wilk test was applied to examine the normality of the continuous variables before inter-group comparison. A Student's *t*-test was used if variables were normally distributed, and a Mann–Whitney *U* test was used to assess non-normal distribution of variables. Spearman's correlation analyses were performed to identify associations between MN CSA and MN mobility related parameters. IBM SPSS Statistics for Mac, Version 23 (IBM Corporation, Armonk, NY, USA) was used for statistical analyses. *p* < 0.05 was set for the level of statistical significance in the two-tailed statistical test.

## Results

### Patient Characteristics

A total of 46 patients (30 women and 16 men) met the inclusion criteria and completed the study. They were equally distributed into the injection (*N* = 23) and surgery (*N* = 23) groups. Baseline demographic and clinical characteristics, including the results of the initial nerve conduction studies, are listed in Table 1. There were no significant inter-group differences in demographic distribution, including mean age, gender, and intervention side. The duration of symptoms was significantly longer in the surgery group. Nerve conduction studies, including distal motor latency, compound muscle action potential, and compound muscle action potential amplitude, showed insignificant differences between the two groups. However, the sensory nerve conduction velocity was slower in the surgery group.

**Table 1:** Baseline characteristics of the study population.

**Variable**	**Injection, *N* = 23**	**Surgery, *N* = 23**	***P*-value**
Mean age (years)	58.2 ± 7.06	58.9 ± 14.4	0.827
Sex (female/male)	15/8	15/8	>0.99
Intervention side (right/left)	17/6	13/10	0.215
Duration of symptom (months)	28.8 ± 53.0	54.4 ± 68.8	0.163
**Nerve conduction study (median nerve)**			
DML (ms)	5.71 ± 1.27	5.88 ± 1.17	0.702
SNCV (m/s)	36.74 ± 7.28	24.34 ± 19.73	**0.039**
CMAP amplitude (mV)	7.36 ± 2.16	6.16 ± 2.78	0.151
SNAP amplitude (μV)	11.05 ± 7.65	7.60 ± 7.41	0.187
BCTQ-SSS	1.66 ± 0.43	1.87 ± 0.57	0.166
BCTQ-FSS	1.52 ± 0.55	2.01 ± 0.75	**0.014**
VAS	5.65 ± 1.41	6.52 ± 1.81	0.076
**Median nerve parameters**			
Cross-sectional area (mm^2^)	15.4 ± 2.7	20.3 ± 7.4	**0.006**
Amplitude (mm)	2.19 ± 1.37	1.80 ± 1.63	0.388
R^2^	0.80 ± 0.20	0.71 ± 0.31	0.259
Curvature	0.57 ± 0.37	0.47 ± 0.42	0.418

AMP, amplitude of median nerve mobility; BCTQ, Boston carpal tunnel questionnaire; CMAP, compound muscle action potential; CSA, cross-sectional area of median nerve at carpal tunnel inlet; DML, distal median nerve latency; FSS, functional status scale; SNAP, sensory nerve action potential; SNCV, sensory nerve conduction velocity; SSS, symptom severity scale; VAS, visual analogue scale. *Significant p values are shown in bold type.

MN CSA were significantly different at baseline; the surgery group had larger CSA. Mobility parameters, including amplitude, R^2^, and curvature, were relatively smaller in the surgery group, but were not significant different (Table 1). This indicated that in the surgery group the MN was more swollen and had less transverse displacement, less functional compliance in response to making a fist, and less transverse sliding ability during the flexion–extension cycle (12). In terms of clinical evaluation, there was no significant difference in the BCTQ-SSS and VAS at baseline; however, the BCTQ-FSS was significantly worse in the surgery group.

### Outcome Measurement

**Figure 3:**
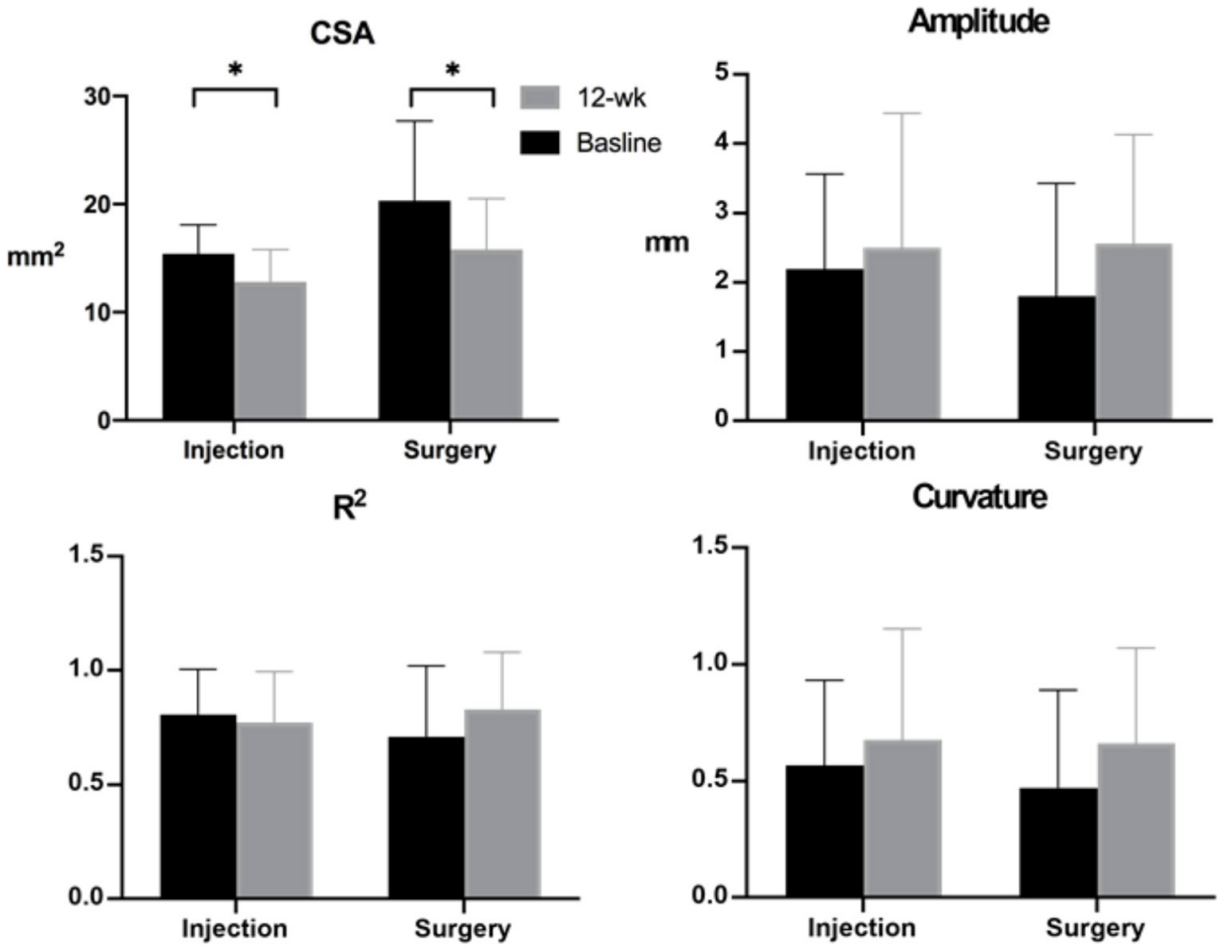
Median nerve cross-sectional area (CSA) and median nerve mobility-related parameters obtained at baseline and 12-week follow-up. Note: *p < 0.05.

After treatment, CSA in both groups showed a significant decrease in size, and the change in CSA did not show inter-group significance (*p* = 0.37). Regarding the amplitude, R^2^, and curvature, all three parameters showed increasing trends but did not reach statistical significance (Figure 3). The change in amplitude, R^2^, and curvature showed insignificant inter-group differences.

**Figure 4:**
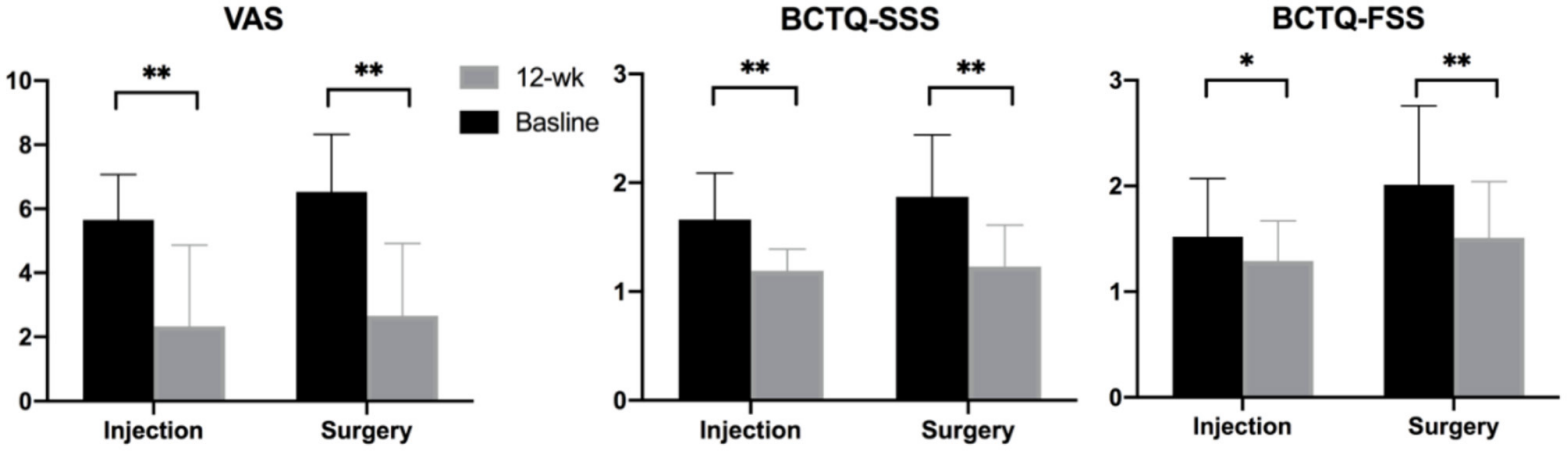
Clinical outcomes measured at baseline and 12-week follow-up. Note: *p < 0.05, **p < 0.001. BCTQ, Boston carpal tunnel questionnaire; FSS, functional status scale; SSS, symptom severity scale; VAS, visual analog scale.

In terms of clinical evaluation, there was no significant difference in the BCTQ-SSS and VAS at baseline; however, the BCTQ-FSS was significantly worse in the surgery group. The BCTQ-SSS, BCTQ-FSS, and VAS scores all showed significant decreases at the 12-week follow-up (Figure 4). Changes in BCTQ-SSS and VAS did not reveal significant inter-group differences, but the change in BCTQ-FSS was significantly larger in the surgery group (SSS, *p* = 0.24; FSS, *p* = 0.046; VAS, *p* = 0.45).

The Spearman correlation between the change in MN mobility parameters and the changes in clinical outcomes found that the amplitude, R^2^, and curvature were significantly correlated in each two-pairs (amplitude vs. R^2^, curvature vs. R^2^, and amplitude vs. curvature), and SSS vs. FSS (all *p* < 0.001). There was no correlation between MN CSA and MN mobility parameters at either baseline or at the 12-week follow-up. In addition, there were no serious complications or adverse effects in either group.

## Discussion

Our study compared MN mobility changes between ultrasound-guided intracarpal injection and CTR for CTS patients, revealing that MN mobility was unchanged by CTR or intracarpal corticosteroid injection. However, both groups showed significant and similar improvements in subjective clinical outcomes and MN CSA.

Although diverse methods for the dynamic measurement of MN mobility have been proposed, the core concept is similar. Most of the dynamic measurements are based on MN displacement during positional changes of the hand from neutral to flexion, with or without a device for positioning (22–26). Existing studies have evaluated the effect of CTR on MN mobility and have shown no significant MN excursion changes in the radial–ulnar plane during the finger flexion cycle (17, 18), but greater displacement in the dorsal–palmar plane (27). Recently, a study compared MN mobility after corticosteroid injection or CTR and found that both interventions improved nerve mobility, while those who underwent surgery had a greater increment of improvement (28). Our study used the previously established tool to differentiate MN mobility in the carpal tunnel (12) by analyzing the MN radio-ulnar plane displacement and its sliding pattern based on its proven ability to classify disease severity, rather than evaluating dorsal–palmar displacement. Moreover, we compared nerve mobility change and clinical outcomes simultaneously to evaluate whether they had prognostic potential.

Several inter-group differences in the baseline parameters reflected heterogeneous disease severity. Patients who were administered the injection tended to have mild to moderate disease, whereas surgery was indicated as conservative treatment for refractory and/or severe disease. In our study, the greater sensory nerve involvement in the surgery group and greater CSA indicated more severe CTS (29–31). Regarding MN mobility, though the baseline inter-group MN mobility difference did not reach statistical significance, a tendency for lower MN mobility was observed in the surgery group, reflecting greater severity (10, 12, 26). Although baseline differences other than MN mobility existed, this study aimed to evaluate whether the changes in MN mobility after the two interventions differed.

Interestingly, the changes in MN mobility were inconsistent with the clinical outcomes. Theoretically, CTR surgery cuts the transverse carpal ligament for decompression, and consequently improves the structural compliance response to force (32). This lessens the constriction for the intracarpal content to move or slide during the flexion–extension cycle, which results in increased MN mobility. By contrast, the idea of perineural injection has been introduced for treating peripheral nerve entrapment. It aims to release the constriction of the peripheral nerve from the adjacent structures by the mechanical force of the needle tip or hydraulic pressure of the injectate (33, 34). Intracarpal corticosteroid injection has no role in alteration of the anatomy of the carpal tunnel space but may decrease MN neural inflammation and lead to a decrease in the MN CSA (13). An *in vitro* study found that triamcinolone acetonide affects transforming growth factor beta signaling regulation in the spermatogonial stem cell transplantation cells of patients with CTS (35), providing an explanation for corticosteroid use in decreased adhesion. However, we did not observe a significant improvement in MN mobility after corticosteroid injection, similar to the findings of a recent study comparing the effects of surgery with steroid injection (19). It is worth noting that previous studies have suggested that ultrasound-guided and landmark-guided corticosteroid injections in CTS produced similar clinical improvements (36, 37). This implies that the corticosteroid injected perineurally to release the adhesion may not be correlated with clinical outcomes and reinforces the fact that decreases in MN mobility caused by tissue adhesion nearby are likely to be a result, rather than a cause of pain in CTS. One might argue that the small volume (2 ml) of injectate used in the present study may separate only the nearby tissue layer surrounding the nerve at the needle tip rather than longitudinally. We could not exclude the possibility of a volume effect, and this may need further investigation.

Because compression and elevated intracarpal pressure are the most accepted pathogenic mechanisms to explain the development of CTS, reducing of the size of the intracarpal content may provide an extended space in the carpal tunnel that allows MN gliding during the flexion–extension cycle. A decrease in MN size was observed in both the surgery and injection groups, but the change in the MN mobility was insignificant. This phenomenon implies that the reversal of neural swelling was not a contributing factor in nerve mobility, regardless of clinical symptom improvement. Furthermore, MN CSA was not correlated with MN mobility parameters at baseline and 12 weeks post-intervention. The trend of a larger CSA and worsening MN mobility may initially be the result of CTS development, in accordance with the previous study by Park et al. (38). Neural swelling and MN mobility were not causally related, and factors such as intracarpal pressure still need to be explored to assess their influence on MN mobility.

The present study has some limitations. First, significant inter-group differences existed at baseline, and whether these differences confounded the results is unclear. Second, this study used a 12-week follow-up period; MN mobility tended to change within 12 weeks, but if the change persisted for a longer period remains undetermined. Third, the evaluation in our study used BCTQ and VAS as clinical outcomes. Performing a post-intervention electrodiagnosis may provide more detailed information regarding MN mobility and its related factors. Fourth, the evaluation of MN mobility in previous studies varies, and the validity of different measurements is unknown, even if the concepts are similar. We assessed MN movement in response to fist, instead of flexed fingers, and wrist simultaneously, and the specified movement during examination may not completely represent nerve mobility. Future studies comparing different evaluation methods, including their reliability and validity, could facilitate the establishment of a standardized method for dynamic nerve mobility evaluation. Last, we focused on the excursion and transverse sliding pattern in the radial–ulnar plane. Concomitant longitudinal excursion during wrist flexion cannot be excluded in two-dimensional ultrasound, and whether the results can be extrapolated to the dorsal-palmar plane, or longitudinal excursion, is unknown.

## Conclusions

In conclusion, both surgery and injection provided significant clinical improvement in the VAS scores and BCTQ subscales. There was no significant improvement of MN mobility and there was no correlation between the change in mobility and amelioration of the clinical condition. Therefore, dynamic MN mobility can be initially assessed to evaluate additional parameters in terms of severity, but its prognostic value for predicting clinical outcomes after interventions in patients with CTS appears to be limited.

## Data Availability Statement

The raw data supporting the conclusions of this article will be made available by the authors, without undue reservation.

## Ethics Statement

The studies involving human participants were reviewed and approved by Taipei Veterans General Hospital. The patients/participants provided their written informed consent to participate in this study.

## Author Contributions

P-CH, I-NL, and J-CW: conceptualization. J-CW: methodology, writing—review and editing, supervision, and funding acquisition. P-CH: software. P-CH and I-NL: validation, data curation, writing—original draft preparation. Y-CH: formal analysis. C-KY: investigation. C-KY and J-CW: resources. Y-CY: visualization and project administration. All authors have read and agreed to the published version of the manuscript.

## Conflict of Interest

The authors declare that the research was conducted in the absence of any commercial or financial relationships that could be construed as a potential conflict of interest.

## Publisher's Note

All claims expressed in this article are solely those of the authors and do not necessarily represent those of their affiliated organizations, or those of the publisher, the editors and the reviewers. Any product that may be evaluated in this article, or claim that may be made by its manufacturer, is not guaranteed or endorsed by the publisher.

## Abbreviations

MN: median nerve
CSA: cross-sectional area
BCTQ: Boston carpal tunnel questionnaire
CTR: carpal tunnel release
VAS: visual analogue scale.
